# Thermally activated delayed fluorescent small molecule sensitized fluorescent polymers with reduced concentration-quenching for efficient electroluminescence

**DOI:** 10.1007/s12200-022-00056-x

**Published:** 2023-03-21

**Authors:** Qin Xue, Mingfang Huo, Guohua Xie

**Affiliations:** 1 Department of Physical Science and Technology, Central China Normal University, Wuhan, 430079 China; 2 Sauvage Center for Molecular Sciences, Hubei Key Lab on Organic and Polymeric Optoelectronic Materials, Department of Chemistry, Wuhan University, Wuhan, 430072 China; 3 Wuhan National Laboratory for Optoelectronics, Huazhong University of Science and Technology, Wuhan, 430074 China

**Keywords:** Thermally activated delayed fluorescence (TADF), Organic light-emitting device (OLED), Sensitization, Energy transfer, Solution process

## Abstract

Thermally activated delayed fluorescence (TADF) small molecule bis-[3-(9,9-dimethyl-9,10-dihydroacridine)-phenyl]-sulfone (*m*-ACSO2) was used as a universal host to sensitize three conventional fluorescent polymers for maximizing the electroluminescent performance. The excitons were utilized via inter-molecular energy transfer and the non-radiative decays were successfully refrained in the condensed states. Therefore, the significant enhancement of the electroluminescent efficiencies was demonstrated. For instance, after doping poly(9,9-dioctylfluorene-co-benzothiadiazole) (F8BT) into *m*-ACSO2, the external quantum efficiency (EQE) was improved by a factor of 17.0 in the solution-processed organic light-emitting device (OLED), as compared with the device with neat F8BT. In terms of the other well-known fluorescent polymers, i.e., poly (para-phenylene vinylene) copolymer (Super Yellow, SY) and poly[2-methoxy-5-(2-ethylhexyloxy)-1,4-phenylenevinylene] (MEH-PPV), their EQEs in the devices were respectively enhanced by 70% and 270%, compared with the reference devices based on the conventional host 1,3-di(9*H*-carbazol-9-yl) benzene (mCP). Besides the improved charge balance in the bipolar TADF host, these were partially ascribed to reduced fluorescence quenching in the mixed films.

**Graphical abstract:**

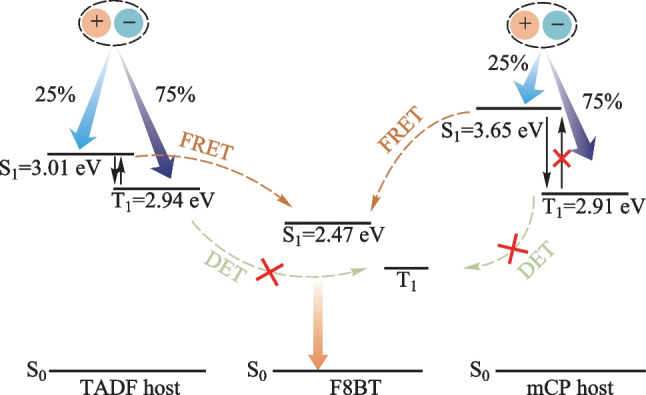

**Supplementary Information:**

The online version contains supplementary material available at 10.1007/s12200-022-00056-x.

## Introduction

The rapid development of organic light-emitting diodes (OLEDs) in the past three decades benefits from the essential innovations of organic materials and devices [1–5]. The conventional fluorescent materials are typically metal-free and easily accessible. Their high color purity and operational stability are ideal for display applications. As for the fluorescent polymers, they are more attractive for solution-processed OLEDs, due to the easily tunable viscosity and excellent morphology. Nevertheless, the electroluminescent (EL) efficiencies of the fluorescent polymer devices fall far behind those of the state-of-the-art OLEDs with the small molecular counterparts featuring either phosphorescence or thermally activated delayed fluorescence (TADF). Due to the spin-orbital coupling in the presence of the heavy metals in the phosphorescent complexes, all the triplet excitons could be utilized in theory which leads to potentially 100% exciton utilization. However, the metal complexes are much more expensive than the purely organic compounds for OLEDs.

**Fig. 1:**
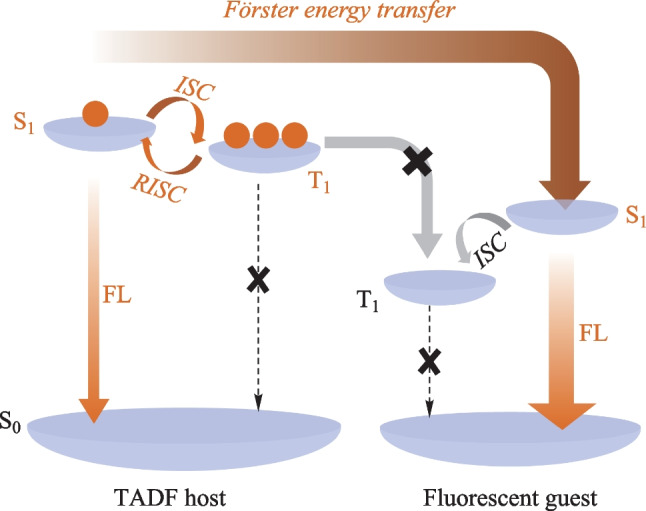
Schematic diagram of energy transfer involved in the system of TADF host and fluorescent guest. S_0_, S_1_, T_1_, FL, ISC and RISC represent the ground state, the lowest singlet state, the lowest triplet state, fluorescence, intersystem crossing, and reverse intersystem crossing, respectively

In the last decade, the brand-new emitters featuring thermally activated delayed fluorescence (TADF), hybridized local and charge-transfer (HLCT), and phosphorescent metal materials have been developed and even used as the sensitizers to maximize the EL performances [6–18]. As shown in Fig. 1, a TADF material could function both as the host and sensitizer to balance charge injection and harvest triplet excitons for the conventional fluorescent emitters. This is one of the most facile ways to improve the EL efficiencies with a simple device architecture [19–25].

**Fig. 2:**
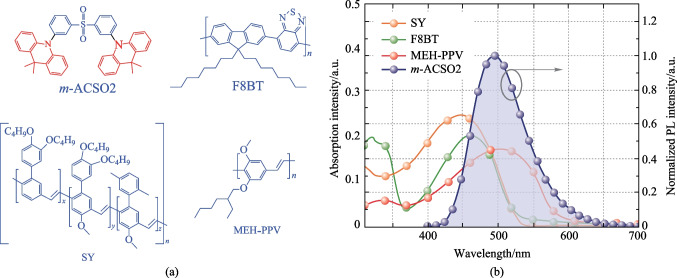
**a** Chemical structures of the molecules used in this study. **b** PL spectrum of *m*-ACSO2 and absorption spectra of SY, F8BT and MEH-PPV in thin films, respectively

Conventionally, the fluorescent OLEDs were fabricated by high-vacuum evaporation with a very low concentration of the fluorescent emitters, e.g., due to the serious fluorescence quenching effect in the condensed states. In this contribution, the fluorescent polymers were mixed with the TADF small molecule bis-[3-(9,9-dimethyl-9,10-dihydroacridine)-phenyl]-sulfone (*m*-ACSO2) with aggregation-enhanced emission [26], which would contribute to high luminous performance in the solution-processed OLEDs. The chemical structures of the molecules used in this study are shown in Fig. 2a, including the polymer guests poly(9,9-dioctylfluorene-co-benzothiadiazole) (F8BT), poly(para-phenylene vinylene) copolymer, Super Yellow (SY), and poly[2-methoxy-5-(2-ethylhexyloxy)-1,4-phenylenevinylene] (MEH-PPV), respectively [27–32]. Eventually, dramatic improvements of the EL performances were realized, e.g., the maximum external quantum efficiencies (EQEs) were enhanced by 17.0 and 6.5 times in the devices with a TADF host and a conventional fluorescent host 1,3-di(9*H*-carbazol-9-yl)benzene (mCP) respectively, as compared with that of the device with the neat F8BT film as the emitting layer. The TADF material as both the host and the sensitizer could harvest triplet excitons via reverse inter-system crossing (RISC), and subsequently transfer them to the fluorescent polymers for light emission. Similarly, the EQEs of the devices employed the polymer guests SY and MEH-PPV doped in *m*-ACSO2, were improved by 70% and 270% respectively, compared with that of the devices with the neat polymers. It is worth mentioning that aggregation-induced quenching of the conventional polymers can be effectively restricted, and aggregation-enhanced emission can be switched on in the mixed solvents triggered by the TADF host.

## Experimental

All the materials involved in this investigation were used as received. The UV–vis absorption spectra were recorded with a Shimadzu UV-2700 spectrophotometer. The photoluminescence spectra were collected by a Hitachi F-4600 fluorescence spectrophotometer. The time-resolved photoluminescence decay curves were recorded by an Edinburgh Instruments spectrometer (FLSP920).

The glass substrates covered with the patterned indium tin oxide (ITO) were cleaned with acetone and ethanol ultrasonic bath, consecutively. Later, the substrates were dried with nitrogen and loaded into a UV-ozone chamber for 20 min. The semiconductive polymer poly(3,4-ethylenedioxythiophene) polystyrene sulfonate (PEDOT:PSS) was spin-coated onto the ITO substrate and then annealed at 120 °C for 10 min. The emitting layers were spin-coating respectively onto PEDOT:PSS, following a annealing process at 50 °C for 10 min. The electron transporting and injecting layers were thermally evaporated in a high vacuum chamber. All the devices were encapsulated with UV-curable resin before taking out the glove-box. The voltage-current-luminance characteristics and the EL spectra were simultaneously measured by a PR735 SpectraScan Spectroradiometer and a Keithley 2400 source meter unit under ambient atmosphere at room temperature.

## Results and discussion

**Fig. 3:**
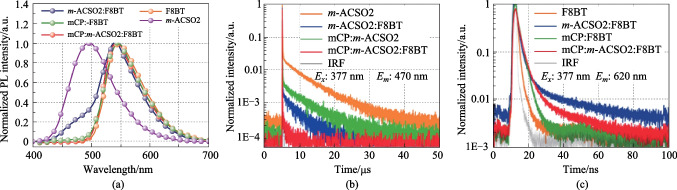
**a** PL spectra of *m*-ACSO2, F8BT and different hosts:F8BT (10 wt%) in the film. **b** Transient PL decay curves of the host-only films and the films with F8BT detected at 470 nm. **c** Transient PL decay curves of different films with F8BT detected at 620 nm at 300 K

Figure 2b shows that the photoluminescence (PL) spectrum of *m*-ACSO2 and the absorption spectra of the three polymers overlap well, which indicates the potentially efficient Fӧrster energy transfer (FRET) [6]. The PL profile of the mixed film *m*-ACSO2:F8BT (10 wt%) is similar to that of the neat film F8BT (see Fig. 3a), except for the residual emission peaking at 470 nm, derived from incomplete energy transfer from *m*-ACSO2 to F8BT. As a reference, in the co-host (mCP:*m*-ACSO2 = 1:1) system, the residual emission peaking at 470 nm was almost quenched, which can be attributed to a faster FRET rate (*k*_ET_ = 4.3 $\times$ 10^8^ s^−1^) of the co-host system than that (*k*_ET_ = 0.9 $\times$ 10^8^ s^−1^) of the TADF host mixed *m*-ACSO2 film (see  Table S1).

As shown in  Table S1, the photoluminescence quantum yields (PLQYs) of the host:SY systems were comparable (81%–94%) with that of the neat SY (86%). For MEH-PPV, the PLQYs of the doped films were almost the same at around 50%, which were sufficiently higher than that of the neat film (14%). In terms of F8BT, the PLQY (21%) of the neat film, was sufficiently lower than those of the doped films. To distinguish the host–guest energy transfer, the transient PL decays were firstly monitored at 470 nm (see Fig. 3b) which mainly excluded the contribution from F8BT. Clearly, the F8BT doped film exhibited the delayed components in the presence of the TADF host *m*-ACSO2. To avoid the influence of *m*-ACSO2 as much as possible, the transient PL decay curves of the different hosts doped with F8BT were detected at 620 nm (see Fig. 3c). Both F8BT and mCP:F8BT exhibited simply mono-exponential decays with the time constants of 1.1 and 2.2 ns respectively, due to their fluorescence nature. In contrast, the transient PL decay curves of *m*-ACSO2:F8BT and co-host:F8BT consisted of the prompt lifetimes of 9.9 and 2.3 ns, and the delayed lifetimes of 2.0 and 1.2 μs respectively. The extended exciton lifetimes indicates the FRET processed from the TADF host to F8BT. Due to the extremely small singlet–triplet gap (Δ*E*_ST_) of *m*-ACSO2, the triplet excitons of *m*-ACSO2 could be easily up-converted to the singlet states through the RISC process at room temperature. Thereafter, the singlet excitons could be transferred to the lowest singlet state (S_1_) of the guest F8BT (see Fig. 1), and boost the exciton utilization efficiency. However, the triplet excitons of the conventional host mCP are non-radiative, leading to inefficient FRET from mCP to F8BT.

**Fig. 4:**
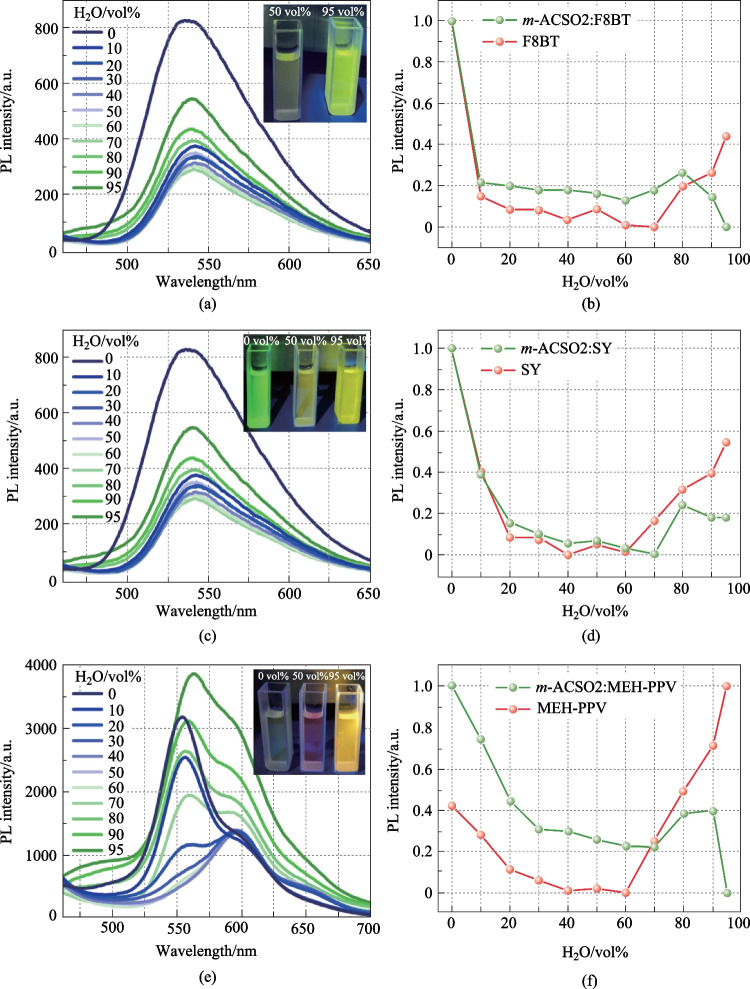
PL spectra of **a** *m*-ACSO2:F8BT, **c** *m*-ACSO2:SY, and **e** *m*-ACSO2:MEH-PPV in THF/H_2_O (10^–5^ mol/L). Inset: Photos of different guests mixed with *m*-ACSO2 in THF (10^–5^ mol/L) with different fractions of H_2_O. PL intensities of **b** F8BT, **d** SY, and **f** MEH-PPV with and without *m*-ACSO2 in THF/H_2_O versus water fractions

Since *m*-ACSO2 exhibited aggregated-enhanced emission [26], the PL experiments of the host–guest system diluted in water/THF with varying water fractions were carried out to further investigate the emissive profiles in the aggregated states. As shown in Fig. 4a, b, and  Fig. S2a, the PL intensity drastically decreased when adding a small amount of water into the THF solution, both for F8BT and *m*-ACSO2:F8BT, which might be attributed to the effect of twisted intra-molecular charge transfer [33]. However, when the water fraction was increased to 80 vol.%, the emission intensity of the solution with *m*-ACSO2:F8BT was dramatically enhanced. In contrast, the emission tended to decrease as for the solution with only F8BT. It is envisaged that the fluorescence quenching of F8BT in the condensed states was easily prohibited by simply doping into the TADF host featuring aggregation-enhanced emission [26]. Likewise, when replacing F8BT with either SY or MEH-PPV, the same tendency was recorded (see Fig. 4c–f). Typically, the TADF sensitizer only transfers the energy to the guest emitters. The sensitizer has little influence on the exciton quenching in the aggregated states. The sensitizer *m*-ACSO2 is powerful in boosting the luminescent intensity of the doped polymers in the aggregated state. This is the unexpectedly added value of the TADF sensitizer *m*-ACSO2, with the characteristic of aggregation-enhanced emission.

**Fig. 5:**
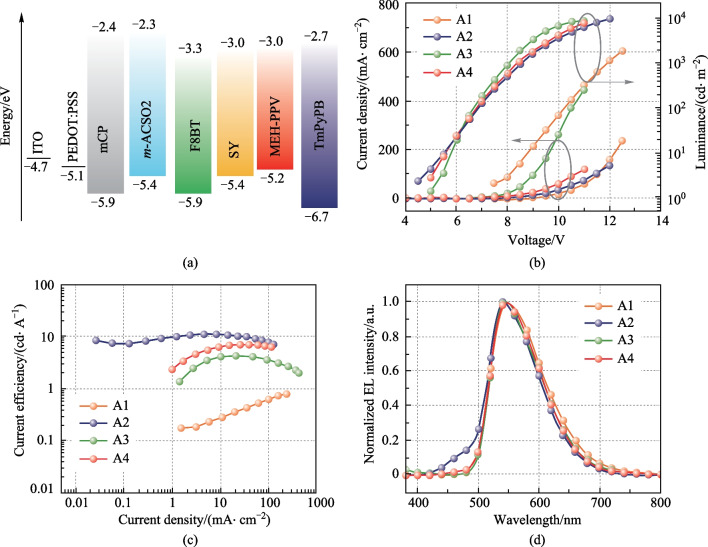
**a** Schematic energy level alignment of the materials used in constructing the devices. **b** Current density–voltage-luminance characteristic curves. **c** Current efficiency versus current density curves. **d** Normalized EL spectra of the devices A1–A4 with F8BT (A1), *m*-ACSO2:F8BT (A2), mCP:F8BT (A3), and mCP:*m*-ACSO2:F8BT (A4) as the emitting layers, respectively

To evaluate the EL performances, the devices were constructed with the architecture (see Fig. 5a) of glass/indium tin oxide (ITO)/poly(3,4-ethylenedioxythiophene):poly (styrenesulfonate) (PEDOT:PSS) (40 nm)/emitting layer (EML) (60 nm)/1,3,5-tri(m-pyridin-3-ylphenyl)benzene (TmPyPB) (40 nm)/8-hydroxyquinolatolithium (Liq) (1 nm)/Al (100 nm), where ITO and Al served as anode and cathode, respectively. PEDOT:PSS was the hole-injecting layer, and TmPyPB and Liq were the electron-transporting layer and the electron-injecting layer, respectively. The key EL parameters of the devices are summarized in Table 1. The device A1 with neat F8BT exhibited an inferior maximum current efficiency of 0.8 cd/A and a low brightness (see Table 1 and Fig. 5b), due to the intensive non-radiative decays in the condensed state. For the devices A2–A4, F8BT was doped into *m*-ACSO2, mCP and the co-host mCP:*m*-ACSO2 with the ratio of 10 wt%, respectively. As we can see from Fig. 5c, the device A2 with the host *m*-ACSO2 demonstrated the superior EL performance with a maximum current efficiency of 11 cd/A, which was 13.8 times higher than that of the device A1 with neat F8BT, and 2.6 times that of the reference device A3, with the host mCP, respectively. Nevertheless, there was some residual emission from the TADF host *m*-ACSO2 in the device A2, which could be reasonably quenched by diluting it with mCP to form a co-host system (see Fig. 5d). In addition, the device A4 based on the co-host presented a maximum current efficiency of 6.9 cd/A, which was 1.6 times higher than that of the reference device A3.

**Table 1:** Comparison of the EL performances of the devices

Device	Host	*V*_10_^a^ /V	EQE_max_^b^ /%	CE_max_^c^ /(cd⋅A^−1^)	PE_max_^d^ /(lm⋅W^−1^)	*λ*_max_^e^ /nm	FWHM^f^ /nm	CIE^g^ (*x*, *y*)	Improvement factor^h^
A1	None	8.8	0.2	0.8	0.2	540	99	(0.43, 0.54)	–
A2	*m*-ACSO2	5.4	3.4	11	4.5	540	92	(0.39, 0.54)	13.8
A3	mCP	5.8	1.3	4.2	1.7	540	93	(0.42, 0.55)	5.3
A4	Co-host	5.6	2.0	6.9	2.5	540	93	(0.42, 0.55)	8.6
B1	None	4.9	2.3	6.3	3.2	560	114	(0.48, 0.51)	–
B2	*m*-ACSO2	4.4	3.9	12.3	7.8	540	101	(0.42, 0.53)	2.0
B3	mCP	4.8	2.2	6.8	3.5	540	102	(0.42, 0.55)	1.1
B4	Co-host	4.7	3.5	11.2	7.2	540	105	(0.41, 0.54)	1.8
C1	None	5.6	0.09	0.09	0.05	640	133	(0.61, 0.38)	–
C2	*m*-ACSO2	5.4	0.33	0.88	0.34	570	82	(0.49, 0.46)	9.7
C3	mCP	5.8	0.10	0.24	0.09	576	89	(0.55, 0.44)	2.7
C4	Co-host	5.4	0.26	0.66	0.33	574	85	(0.51, 0.45)	7.3

^a^Driving voltage at luminance of 10 cd/m^2^. ^b^Maximum external quantum efficiency (EQE_max_), ^c^maximum current efficiency (CE_max_), ^d^Maximum power efficiency (PE_max_). ^e^Peak emission wavelength of the EL spectra. ^f^Full-width at half-maximum of the EL spectra. ^g^Commission Internationale de I'Eclairage (CIE) coordinates. ^h^The enhanced factor of CE_max_ of the device with the TADF host *m*-ACSO2, compared with the non-doped devices

**Fig. 6:**
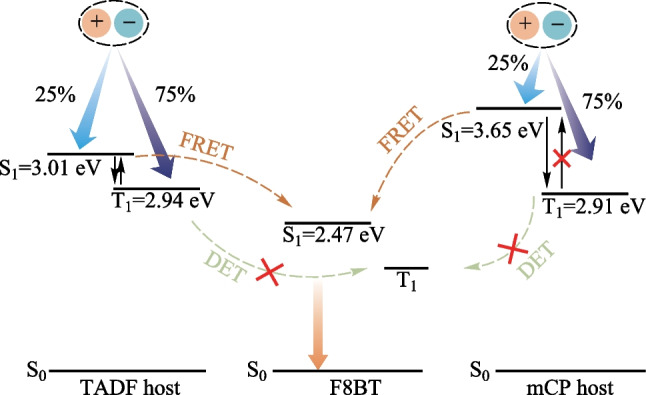
Schematic illustration of energy transfer respectively from the TADF host and the conventional fluorescent host mCP to the fluorescent polymer guest F8BT

In principle, the TADF host *m*-ACSO2 possesses the shallower highest occupied molecular orbital (HOMO) level than that of mCP, which is beneficial for hole injection from PEDOT:PSS (see Fig. 5a). This resulted in the lower turn-on voltage of the device A2. In contrast to the hole-dominated host mCP, the inherently bipolar nature of *m*-ACSO2 would accelerate charge balance in the emissive zone, which is crucial for improving radiative recombination. Last but not the least, the TADF host *m*-ACSO2 can take the advantage of triplet excitons up-conversion through RISC process. Nevertheless, only 25% excitons can be theoretically utilized if, the conventional mCP host was used in such device architecture (see Fig. 6).

To assure the generality of the TADF host *m*-ACSO2 as a good sensitizer for the fluorescent polymer guests, two additional guests were studied. Figures S1a and S1b suggest that the emission peaks of the doped films based on SY and MEH-PPV, i.e., 540 and 580 nm, respectively, were both slightly blue-shifted as compared with those of the neat films. The time constants (see Table S1) observed at 620 nm of the films doped with the conventional host mCP, co-host and *m*-ACSO2 were increasing in sequence. As indicated in Table S1, all FRET rate constants of (*k*_ET_) are higher than the corresponding radiative rate constants (${k}_{\text{r}}^{\mathrm{S}}$), suggesting efficient FRET from host to guest.

Likewise, similar architectures of the devices were constructed to compare the EL performances for different polymers. The device series of B1–B4 for the yellow polymer SY and C1–C4 for the red polymer MEH-PPV are similar to those of A1–A4. Coincidentally, the EL performances were significantly improved by mixing *m*-ACSO2 with the polymers. For instance, the maximum current efficiencies of the devices B2 and C2 were respectively enhanced by 80% and 267% (see Fig. S3 and Table 1), as compared with those of the reference devices B3 and C3 based on the conventional fluorescent host mCP. These results match well with the analysis mentioned previously. The EQEs summarized in Table 1 are basically consistent with the PLQYs shown in Table S1, except for the device with the fluorescent host mCP. Although the polymers doped in mCP achieved significantly improved PLQYs, the device performances were inferior to those with the TADF sensitizer since the polymers could not harvest triplet excitons from the fluorescent host mCP.

## Conclusions

In conclusion, we demonstrated tremendous improvements of the EL performances by introducing a TADF host with aggregation-enhanced emission to sensitize the fluorescent polymers, which overturned the quenching effect of the polymers in the condensed states. For the green fluorescent polymer F8BT, the current efficiency, the power efficiency, and the external quantum efficiency were enhanced by factors of 13.8, 22.5 and 17.0, respectively, compared with those of the non-doped device. These benefits were ascribed to the enhanced balance of charge carriers and energy transfer, as well as restricted fluorescence quenching in the presence of the multi-functional TADF host. The findings through this investigation are supposed to be universal and applicable to many other fluorescent emitters.

## Supplementary Information

Below is the link to the electronic supplementary material.

Supplementary file1 (PDF 1450 KB)

## Data Availability

Data are available by request from the authors.

## References

[1] Tang C, VanSlyke S (1987). Organic electroluminescent diodes. Appl. Phys. Lett..

[2] Baldo M, O’Brien D, You Y, Shoustikov A, Sibley S, Thompson M, Forrest S (1998). Highly efficient phosphorescent emission from organic electroluminescent devices. Nature.

[3] Baldo M, Lamansky S, Burrows P, Thompson M, Forrest S (1999). Very high-efficiency green organic light-emitting devices based on electrophosphorescence. Appl. Phys. Lett..

[4] Adachi C, Baldo M, Thompson M, Forrest S (2001). Nearly 100% internal phosphorescence efficiency in an organic light-emitting device. J. Appl. Phys..

[5] Uoyama H, Goushi K, Shizu K, Nomura H, Adachi C (2012). Highly efficient organic light-emitting diodes from delayed fluorescence. Nature.

[6] Nakanotani H, Higuchi T, Furukawa T, Masui K, Morimoto K, Numata M, Tanaka H, Sagara Y, Yasuda T, Adachi C (2014). High-efficiency organic light-emitting diodes with fluorescent emitters. Nat. Commun..

[7] Tao Y, Yuan K, Chen T, Xu P, Li H, Chen R, Zheng C, Zhang L, Huang W (2014). Thermally activated delayed fluorescence materials towards the breakthrough of organoelectronics. Adv. Mater..

[8] Zhang D, Duan L, Li C, Li Y, Li H, Zhang D, Qiu Y (2014). High-efficiency fluorescent organic light-emitting devices using sensitizing hosts with a small singlet-triplet exchange energy. Adv. Mater..

[9] Higuchi T, Nakanotani H, Adachi C (2015). High-efficiency white organic light-emitting diodes based on a blue thermally activated delayed fluorescent emitter combined with green and red fluorescent emitters. Adv. Mater..

[10] Lee I, Song W, Lee J, Hwang SH (2015). High efficiency blue fluorescent organic light-emitting diodes using a conventional blue fluorescent emitter. J. Mater. Chem. C Mater. Opt. Electron. Devices.

[11] Liu XK, Chen Z, Qing J, Zhang WJ, Wu B, Tam HL, Zhu F, Zhang XH, Lee CS (2015). Remanagement of singlet and triplet excitons in single-emissive-layer hybrid white organic light-emitting devices using thermally activated delayed fluorescent blue exciplex. Adv. Mater..

[12] Marian C (2016). Mechanism of the triplet-to-singlet upconversion in the assistant dopant ACRXTN. J. Phys. Chem. C.

[13] Chen D, Cai X, Li XL, He Z, Cai C, Chen D, Su SJ (2017). Efficient solution-processed red all-fluorescent organic light-emitting diodes employing thermally activated delayed fluorescence materials as assistant hosts: molecular design strategy and exciton dynamic analysis. J. Mater. Chem. C Mater. Opt. Electron. Devices.

[14] Chen L, Zhang S, Li H, Chen R, Jin L, Yuan K, Li H, Lu P, Yang B, Huang W (2018). Breaking the efficiency limit of fluorescent OLEDs by hybridized local and charge-transfer host materials. J. Phys. Chem. Lett..

[15] Zhang D, Song X, Cai M, Duan L (2018). Blocking energy-loss pathways for ideal fluorescent organic light-emitting diodes with thermally activated delayed fluorescent sensitizers. Adv. Mater..

[16] Kim HG, Kim KH, Moon CK, Kim JJ (2017). Harnessing triplet excited states by fluorescent dopant utilizing codoped phosphorescent dopant in exciplex host for efficient fluorescent organic light emitting diodes. Adv. Opt. Mater..

[17] Kim HG, Kim KH, Kim JJ (2017). Highly efficient, conventional, fluorescent organic light-emitting diodes with extended lifetime. Adv. Mater..

[18] Jou JH, Fu SC, An CC, Shyue JJ, Chin CL, He ZK (2017). High efficiency yellow organic light-emitting diodes with a solution-process feasible iridium based emitter. J. Mater. Chem. C Mater. Opt. Electron. Devices.

[19] Xue J, Liang Q, Zhang Y, Zhang R, Duan L, Qiao J (2017). High-efficiency near-infrared fluorescent organic light-emitting diodes with small efficiency roll-off: a combined design from emitters to devices. Adv. Funct. Mater..

[20] Ahn DH, Jeong JH, Song J, Lee JY, Kwon JH (2018). Highly efficient deep blue fluorescent organic light-emitting diodes boosted by thermally activated delayed fluorescence sensitization. ACS Appl. Mater. Interfaces.

[21] Han S, Lee J (2018). Spatial separation of sensitizer and fluorescent emitter for high quantum efficiency in hyperfluorescent organic light-emitting diodes. J. Mater. Chem. C Mater. Opt. Electron. Devices.

[22] Furukawa T, Nakanotani H, Inoue M, Adachi C (2015). Dual enhancement of electroluminescence efficiency and operational stability by rapid upconversion of triplet excitons in OLEDs. Sci. Rep..

[23] Song W, Lee I, Lee JY (2015). Host engineering for high quantum efficiency blue and white fluorescent organic light-emitting diodes. Adv. Mater..

[24] Wu Z, Wang Q, Yu L, Chen J, Qiao X, Ahamad T, Alshehri SM, Yang C, Ma D (2016). Managing excitons and charges for high-performance fluorescent white organic light-emitting diodes. ACS Appl. Mater. Interfaces.

[25] Wu Z, Yu L, Zhou X, Guo Q, Luo J, Qiao X, Yang D, Chen J, Yang C, Ma D (2016). Management of singlet and triplet excitons: a universal approach to high-efficiency all fluorescent WOLEDs with reduced efficiency roll-off using a conventional fluorescent emitter. Adv. Opt. Mater..

[26] Wu K, Wang Z, Zhan L, Zhong C, Gong S, Xie G, Yang C (2018). Realizing highly efficient solution-processed homojunction-like sky-blue OLEDs by using thermally activated delayed fluorescent emitters featuring an aggregation-induced emission property. J. Phys. Chem. Lett..

[27] Demir N, Oner I, Varlikli C, Ozsoy C, Zafer C (2015). Efficiency enhancement in a single emission layer yellow organic light emitting device: contribution of CIS/ZnS quantum dot. Thin Solid Films.

[28] Liu F, Chen Z, Du X, Zeng Q, Ji T, Cheng Z, Jin G, Yang B (2016). High efficiency aqueous-processed MEH-PPV/CdTe hybrid solar cells with a PCE of 4.20%. J. Mater. Chem. A Mater. Energy Sustain..

[29] Bolink H, Coronado E, Orozco J, Sessolo M (2009). Efficient polymer light-emitting diode using air-stable metal oxides as electrodes. Adv. Mater..

[30] Kim YH, Han TH, Cho H, Min SY, Lee CL, Lee TW (2014). Polyethylene imine as an ideal interlayer for highly efficient inverted polymer light-emitting diodes. Adv. Funct. Mater..

[31] Yin X, Xie G, Peng Y, Wang B, Chen T, Li S, Zhang W, Wang L, Yang C (2017). Self-doping cathode interfacial material simultaneously enabling high electron mobility and powerful work function tunability for high-efficiency all-solution-processed polymer light-emitting diodes. Adv. Funct. Mater..

[32] Yin X, Xie G, Zhou T, Xiang Y, Wu K, Qin J, Yang C (2016). Simple pyridine hydrochlorides as bifunctional electron injection and transport materials for high-performance all-solution-processed organic light emitting diodes. J. Mater. Chem. C Mater. Opt. Electron. Devices.

[33] Sasaki S, Drummen G, Konishi G (2016). Recent advances in twisted intramolecular charge transfer (TICT) fluorescence and related phenomena in materials chemistry. J. Mater. Chem. C Mater. Opt. Electron. Devices.

