# Supplementary material for: Thermally activated delayed fluorescent small molecule sensitized fluorescent polymers with reduced concentration-quenching for efficient electroluminescence
Source: Front Optoelectron. 2023 Mar 21;16(1):2. doi: 10.1007/s12200-022-00056-x (PMC10027968; doi:10.1007/s12200-022-00056-x)
Supplement: Supplementary file 1 — Supplementary file1 (PDF 1450 KB) [file 12200_2022_56_MOESM1_ESM.pdf]

Supporting Information

Thermally Activated Delayed Fluorescent Small Molecule Sensitized Fluorescent Polymers with Reduced Concentration-Quenching for Efficient Electroluminescence

*Qin Xue[1], Mingfang Huo[1], and Guohua Xie[2,3]**

[1]Department of Physical Science and Technology, Central China Normal University, Wuhan 430079, China

[2]Sauvage Center for Molecular Sciences, Hubei Key Lab on Organic and Polymeric Optoelectronic Materials, Department of Chemistry, Wuhan University, Wuhan 430072, China

[3]Wuhan National Laboratory for Optoelectronics, Huazhong University of Science and Technology, Wuhan 430074, China

*Corresponding email addresses: guohua.xie@whu.edu.cn

Experimental Section

General information: All the materials except *m*-ACSO2 synthesized in our lab according to the literature were purchased from commercial sources and used as received. The absorption and PL spectra were measured by a Shimadzu UV-2700 UV-VIS spectrophotometer and a Hitachi F-4600 fluorescence spectrophotometer, respectively. The transient PL spectra were measured by single photon counting spectrometer on Edinburgh Instruments (FLS920) with an excitation light source of Picosecond Pulsed UV-LASTER (LASTER377).

OLED fabrication and characterization: The pre-patterned indium tin oxide (ITO) glass substrates were cleaned consecutively in ultrasonic acetone ethanol baths. Afterwards, the substrates were dried with N_2 and then loaded into a UV-ozone chamber. After UV-ozone treatment for 20 min, a layer of PEDOT:PSS was spin-coated on the ITO substrate as the hole-injecting layer and then annealed at 120 ^{o}C for 10 min inside the N_2-filled glove-box. The emitter layer was prepared by spin-coating directly on PEDOT:PSS and then annealed at 50 ^{o}C for 10 min. The electron transporting and injecting materials and the cathode were thermally evaporated onto the emitter layer in a vacuum chamber. Before being taken out of the glove-box, the devices were encapsulated with UV-curable epoxy. The voltage-current-luminance characteristics and the EL spectra were simultaneously measured with a PR735 SpectraScan Spectroradiometer and a Keithley 2400 source meter unit under ambient atmosphere at room temperature.

Table S1. Photophysical data of the spin-coat films.

Films	τ_p (ns)	τ_d (μs)	Φ_p (%)	Φ_d (%)	k_p (10^8 s^{-1})	k_d (10^5 s^{-1})	k_{ET} (10^8 s^{-1})	k_r^S (10^8 s^{-1})
m-ACSO2	67.6	6	17	59	0.1	1.7	-	0.03
co-host	63.5	5.8	18	47	0.1	1.7	-	0.03
F8BT	1.1	-	21	-	9.1	-	-	1.9
m-ACSO2:F8BT	9.9	2.0	34	22	1.0	5.0	0.9	0.3
*m*CP:F8BT	2.2	-	82	-	4.5	-		3.7
co-host:F8BT	2.3	1.2	57	14	4.3	8.3	4.3	2.5
SY	1.6	-	86	-	6.3	-	-	5.3
m-ACSO2:SY	10.3	2.0	66	15	1.0	5.0	0.8	0.6
*m*CP:SY	1.0	-	91	-	10.0	-		9.1
co-host:SY	6.8	1.4	88	6	1.5	7.1	1.5	1.2
MEH-PPV	0.7	-	14	-	14.3	-	-	2.0
m-ACSO2:MEH-PPV	1.1	0.0092	32	18	9.1	1087.0	8.9	2.9
*m*CP:MEH-PPV	0.8	-	50	-	12.5	-		6.3
co-host:MEH-PPV	0.8	0.0056	38	16	12.5	1785.7	12.5	4.7

The rate constants of the spin-coat films are calculated by using the equations described as below:

$$k_p = \frac{1}{\tau_p} \qquad (1)$$

$$k_d = \frac{1}{\tau_d} \quad (2)$$

$$k_r^{\mathrm{S}} = \Phi_p k_p \quad (3)$$

$$k_{nr}^{\mathrm{S}} = (\frac{\Phi_p}{\Phi} - \Phi_p)k_p \quad (4)$$

$$k_{nr}^{\mathrm{T}} = k_d - \Phi_p k_{RISC} \quad (5)$$

$$k_{ISC} = (1 - \frac{\Phi_p}{\Phi})k_p \quad (6)$$

$$k_{RISC} = \frac{k_p k_d \Phi_d}{k_{ISC}\Phi_p} \quad (7)$$

Where τ_p and τ_d are the transient decay time constants of the prompt component and delayed component, respectively. Φ_p and Φ_d are the prompt and delayed components of the PL quantum efficiency, respectively.

For *m*-ACSO2 and co-host films without guests, $k_{p,H}$ can be described as

$$k_{p,H} = k_r^{\mathrm{S}} + k_{ISC} + k_{nr}^{\mathrm{S}} \quad (8)$$

For the host-guest doped films, $k_{p,HG}$ can be described as

$$k_{p,HG} = k_r^{\mathrm{S}} + k_{ISC} + k_{nr}^{\mathrm{S}} + k_{ET} \quad (9)$$

Where k_{ET} is the rate constant of Förster energy transfer.

Therefore, k_{ET} can be describe as

$$k_{ET} = k_{p,HG} - k_{p,H} = \frac{1}{\tau_{p,HG}} - \frac{1}{\tau_{p,H}} \quad (10)$$

Where $\tau_{p,HG}$ is the prompt fluorescent lifetime of the films with the guests and $\tau_{p,H}$ is the prompt fluorescent lifetime of the host films. [1-5]

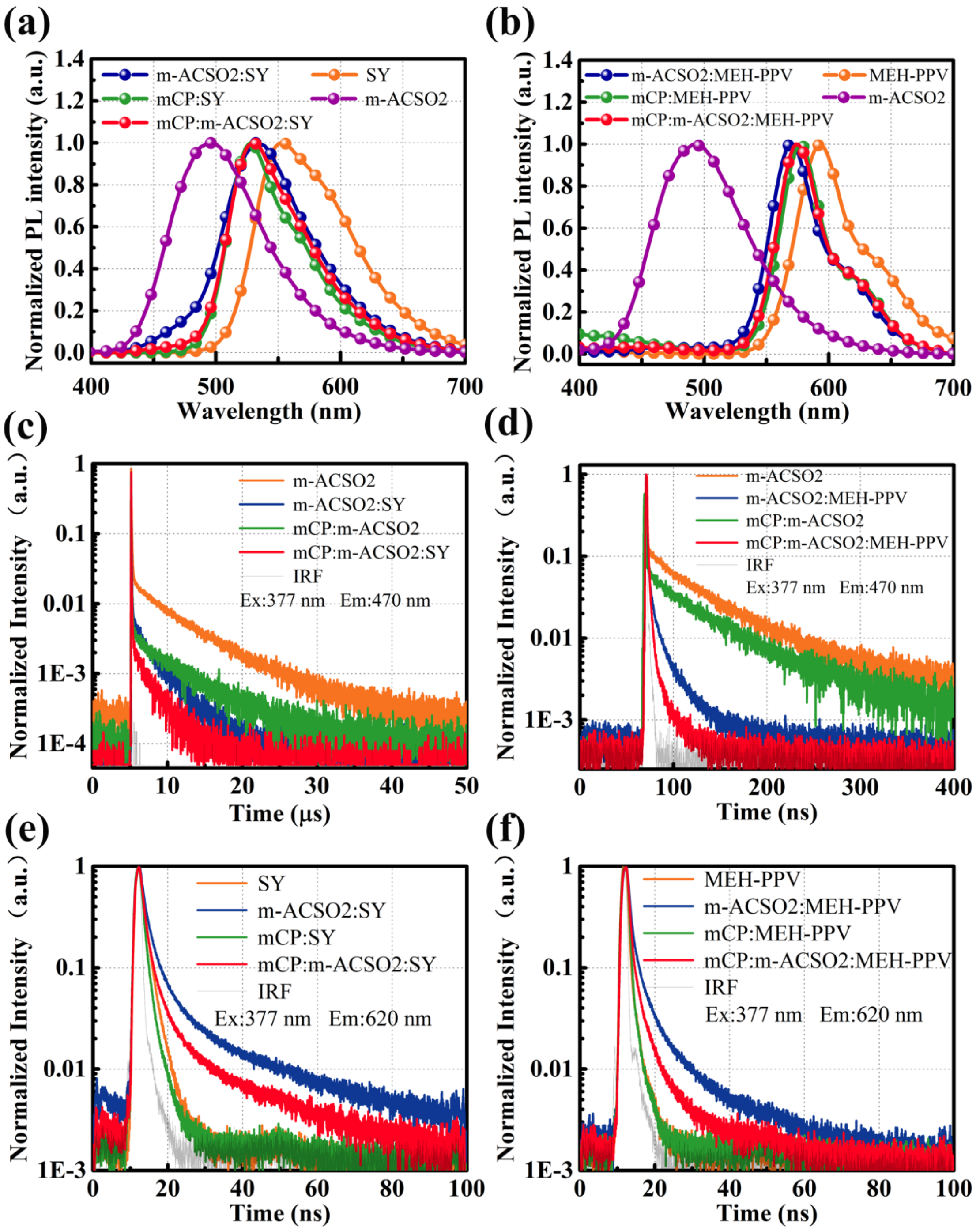

Figure S1. Normalized PL spectra of the doped films based on (a) SY and (b) MEH-PPV. Transient PL decay curves of (c) SY and (d) MEH-PPV in different films detected at 470 nm, respectively. Transient PL decay curves of (e) SY and (f) MEH-PPV in different films detected at 620 nm, respectively.

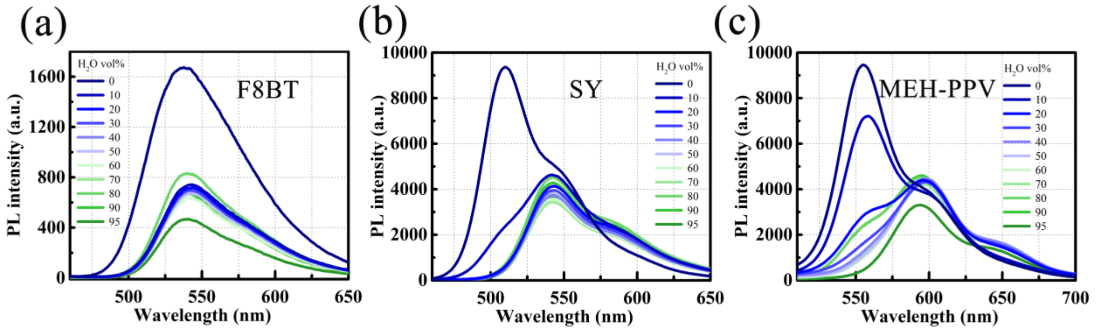

Figure S2. PL emission spectra of (a) F8BT, (b) SY and (c) MEH-PPV in THF/H_2O (10^{-5} M) with different water fractions.

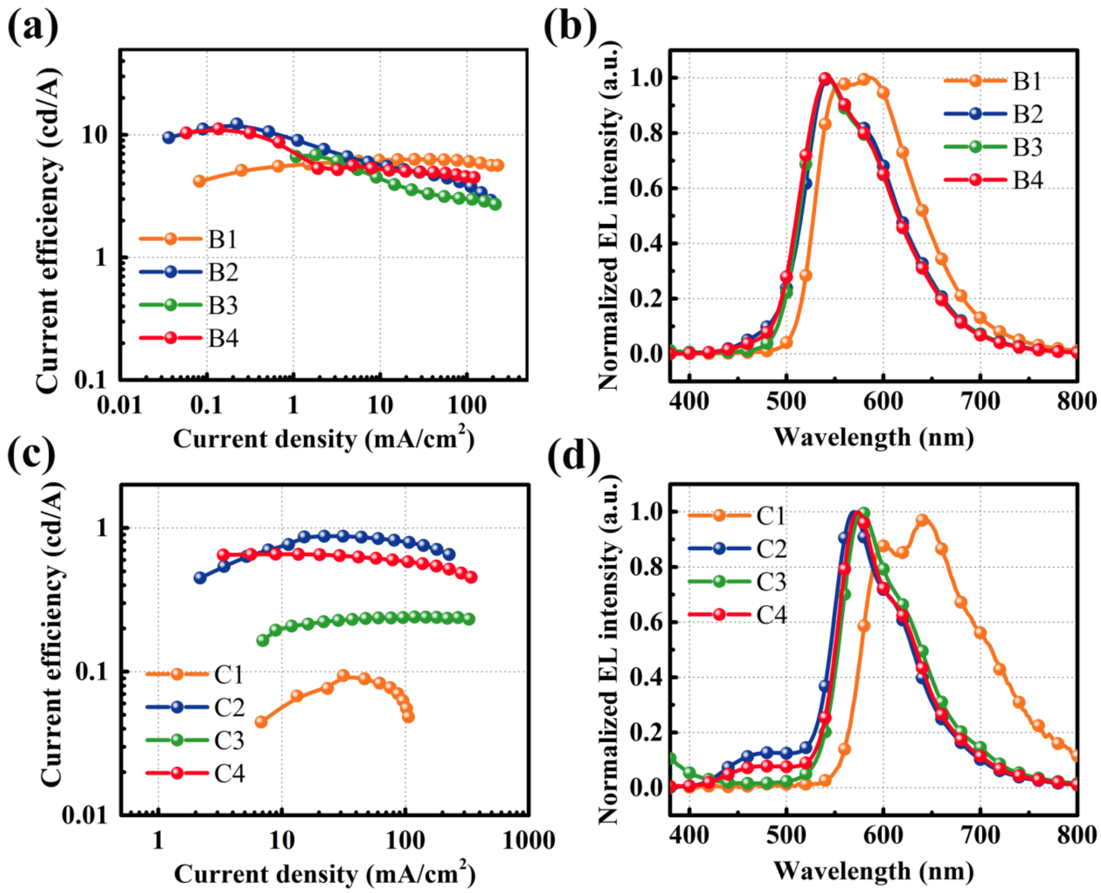

Figure S3. (a) Current efficiency versus current density curves and (b) normalized EL spectra of the devices B1–B4. (c) Current efficiency versus current density curves and (d) normalized EL spectra of the devices C1–C4.

Reference

1 Kenichi Goushi, Kou Yoshida, Keigo Sato and Chihaya Adachi, *Nat. Photonics*, **2012**, 6, 253.

2 Hiroki Uoyama, Kenichi Goushi, Katsuyuki Shizu, Hiroko Nomura and Chihaya Adachi, *Nature*, **2012**, 492, 234.

3 Qisheng Zhang, Hirokazu Kuwabara, William J. Potscavage, Shuping Huang, Yasuhiro Hatae, Takumi Shibata and Chihaya Adachi, *J. Am. Chem. Soc.*, **2014**, 136, 18070-18081.

4 Hiroyuki Tanaka, Katsuyuki Shizu, Jiyoung Lee and Chihaya Adachi, *J. Phys. Chem. C*, **2015**, 119, 2948-2955.

5 Dongjun Chen, Xinyi Cai, Xiang-Long Li, Zuozheng He, Chengsong Cai, Dongcheng Chen and Shi-Jian Su, *J. Mater. Chem. C*, **2017**, 5, 5223-5231.
